# Imaging features of fungal infection in immuno-suppressed patients in a local ward outbreak

**DOI:** 10.2349/biij.2.2.e21

**Published:** 2006-04-01

**Authors:** S Ahmad Sarji, WA Wan Abdullah, ML Wastie

**Affiliations:** 1 Department of Biomedical Imaging, Faculty of Medicine, University of Malaya, Kuala Lumpur, Malaysia; 2 Department of Paediatrics, University of Malaya, Kuala Lumpur, Malaysia

**Keywords:** Leukaemia, fungal infection, air contamination, Aspergillosis

## Abstract

**Purpose of study:**

To examine the role of imaging in diagnosing and assessing fungal infections in paediatric patients undergoing chemotherapy in a facility, which had high fungal air contamination due to adjacent building construction work.

**Materials and method:**

Nineteen patients aged five months to 12 years with various malignancies, mainly leukaemia, along with probable fungal infection were referred for imaging over a period of 12 months. The imaging findings from their CT and chest radiographs were reviewed by two radiologists and correlated with the clinical findings. Blood culture and/or biopsy of relevant lesions were performed for all patients.

**Results:**

Fungus was positively isolated in 11 out of 19 patients, but the remaining patients clinically had fungal infection. The most common species isolated was *Candida sp.* (five patients), followed by *Aspergillus sp.* The most common site of fungal infection was the lungs (10 out of 19 patients), where consolidation or cavitating nodules were seen on CT or the plain chest radiograph. One patient developed pulmonary artery aneurysm as a complication. The other sites affected were the intra-abdominal organs (liver, kidneys, and spleen) and the paranasal sinuses, shown on CT. Two patients with clinical evidence of infection and *Candida sp.* isolated from their blood, however, showed no abnormal findings on imaging.

**Conclusion:**

Early diagnosis of fungal infections in oncology patients undergoing chemotherapy is important, but diagnosis may be difficult through imaging because of the non-specific changes and the presence of abnormalities from the underlying disease. Even if a specific diagnosis cannot be reached, imaging is useful to monitor response to treatment and detect complications.

## INTRODUCTION

Fungal infections are a major cause of morbidity and mortality in patients with haematological and other malignancies. Aspergillus and Candida species are the most common fungal pathogens, although other unusual fungi are also becoming recognized. Filamentous fungi, such as, Aspergillus are soil inhabitants, but the airborne fungal contamination may increase if the soil is disturbed. An outbreak of fungal infection in a ward for children with leukaemia and other malignancies undergoing chemotherapy is reported. This infection was aggravated by the soil being disturbed by adjacent building construction. Early diagnosis and treatment of fungal infection is important, but imaging may not provide diagnosis because of the non-specific changes and the presence of abnormalities from the underlying disease.

## MATERIALS AND METHOD

During the period 2002-2003, 19 children in a paediatric ward were diagnosed with a proven or probable fungal infection. This ward caters mainly to children with leukaemia; 15 children had leukaemia, one had a medulloblastoma, one had a sacrococcygeal tumour, and two had neuroblastoma.

Air quality tests for fungal air contaminants were performed in different parts of the ward.

Plain chest radiography and CT were used for imaging as the majority of patients had clinical signs of respiratory tract and pulmonary involvement. The findings on chest radiograph and CT were reviewed to see how imaging could assist in the diagnosis of fungal infection.

## RESULTS

A summary of patient data and findings is presented in Table 1. As the increased number of fungal infections was thought to be due to soil disturbance at the adjacent building work site, air quality tests for fungal air contaminants were performed. The measurement was in colony forming units/m3 (cfu/m3). Measurement obtained from the treatment room was 659 cfu/m3, one of the single bedded rooms was 206 cfu/m3, and the paediatric day care unit was 218 cfu/m3.

**Table 1:** Patient details. * Prophylaxis consisted of itraconazole and granulocyte colony stimulating factor (GCSF).

**Case**	**Age/Sex**	**Disease**	Prophylaxis*	**Sites**	**Fungus**	**Outcome**	**Radiological features**
1	4/F	AML	No	Sinuses	Mucor species	Resolved	CT – Sinus disease with destruction of medial wall of right antrum .
2	1/F	ALL	No	Lungs kidneys	Aspergillus	Resolved	CXR – Consolidation of left lower lobe. CT – Cavitation in left lower lobe. Large kidneys with many low attenuation areas.
3	4/M	AML	No	Lungs	Presumed resolved after Amphotericin	Succumbed to klebsiella sepsis	CXR – Lung nodules which subsequently cavitated
4	6 months/M	AML	No	Lungs, liver, oesophagus	Candida from oesophagus	Died	No abnormal radiological features
5	6/F	ALL	No	Liver, spleen, kidneys, skin	Unidentified fungal elements from skin	Died	CT – Multiple low attentuation areas in liver, spleen and kidneys
6	1/F	AML	Yes	Lungs	None isolated but clinical course suggested fungal infection	Died	CXR – Bilateral lung consolidation
7	7/M	ALL	Yes	Blood, liver	Candida tropicalis	Died	No abnormal radiological features
8	3/M	ALL	No	Liver, spleen,	None isolated	Resolved	CT – Hypodense lesions in liver and spleen
9	12/M	AML	Yes	Blood, sinuses, lungs	Fusarium oxysporon from blood	Died	CT – Sinus disease in antra. Small lung nodules and consolidation which cavitated.
10	2/F	ALL	Yes	Blood	Candida pelliculosa	Cured	No abnormal radiological features
11	1/F	Medulloblastoma	No	Lungs	Candida albicans. Trichosporon species	Died	CXR – Bilateral upper lobe consolidation
12	5/F	Neuroblastoma	Yes	Lungs	Presumed	Cured	CXR – Bilateral lung consolidation
13	3/M	ALL	Yes	Sinuses	None isolated but presumed	Remission	CT – Mucosal thickening both antra. With destruction of lateral wall right maxillary antrum
14	7/M	ALL	Yes	Sinuses	None isolated	Remission	CT – Pansinusitis. Hepatosplenomegaly. Cerebral venous thrombosis. Small enhancing cerebral lesions
15	9/F	AML	Yes	Lungs	Aspergillus candida tropicalis	Delayed chemotherapy	CXR and CT – Lung nodules which cavitated. Mycotic pulmonary artery aneurysm
16	3/M	Sacrococcygeal tumour	No	Blood	Paecilomyces	Died	CT – Nodules increasing on anti fungal treatment
17	3/M	ALL	Yes	Lungs	None isolated	Died	CXR – Consolidation right lower and left upper lobes
18	11/F	AML	Yes	Lungs	None isolated	Died	CXR – Consolidation right lung
19	5 months/M	Neuroblastoma	No	Blood	Candida	Delayed chemotherapy	CT – Adrenal tumour. Hypodense liver lesions thought to be metastases

The species of fungi isolated included Aspergillus, Fusarium, Chrysosporium, Mycelium, Monilia, and Phaecilomyces.

## DISCUSSION

Patients with haematological malignancies suffer from several deficits in host defence, which make the patient susceptible to fungal infections, especially Candida and Aspergillus species.

The opportunistic yeasts of the Candida species are endogenous flora that can gain access to the bloodstream, usually through the bowel [1]. There may be a superficial infection, such as, oesophageal candidiasis, and the disease may be disseminated to visceral sites. Candida also infects the lungs, causing a miliary nodular pattern [2]. The diagnosis can be difficult because other opportunistic infections may be present and there may be superficial incidental colonisation by Candida. Most patients with Candida infection have widespread systemic involvement, and Candida pneumonia without systemic involvement is rare [3,4]. In our study, Candida was isolated in five patients, but not all of them had abnormalities on imaging. In fact, two patients showed no abnormal findings on imaging.

**Figure 1:**
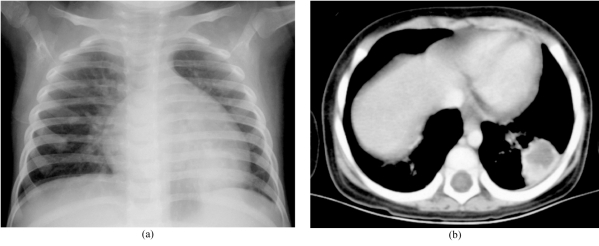
An 18-month-old girl with leukaemia and Aspergillus isolated from the lung. (a) Chest radiograph showed a round opacity behind the heart. (b) CT revealed a cavitating nodule in the left lower lobe.

The spores of Aspergillus species enter the body commonly through the sinuses or the respiratory tract. Aspergillus infects the airways, resulting in bronchopneumonia, but in the early stages of the disease the chest radiograph may be normal. As the disease progresses, a nodular appearance or patchy consolidation may be evident. Aspergillus is angioinvasive and its most frequent appearance is a single area or multiple areas of rounded consolidation that may cavitate (Figure 1). The areas of consolidation are infarcts resulting from vascular invasion by Aspergillus. Cavitation is thought to be a favourable sign, indicating a significant defence response [5]. Of the four patients with cavitating lung nodules, Aspergillus was isolated in two.

**Figure 2:**
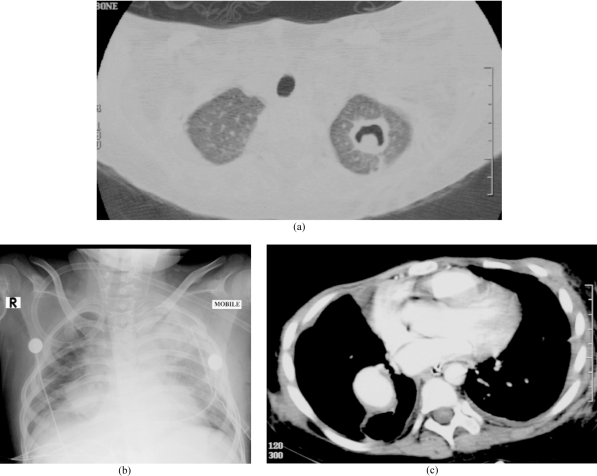
A 10-year-old girl with leukemia and biopsy proven Aspergillus infection of the lung. She later developed a mycotic aneurysm in the chest. (a) CT showed a cavity with a “crescent sign” in keeping with an Aspergilloma. The lesion was excised. (b) Chest radiograph two months later showed a round opacity in the right lung. (c) CT revealed an aneurysm of the lower lobe branch of the right pulmonary artery.

Because of the tendency of the organism to invade pulmonary blood vessels, mycotic aneurysms of the pulmonary artery may occur, which can be mistaken on the chest radiograph for areas of consolidation (Figure 2). Extension of the pulmonary parenchymal lesion to involve the mediastinum can occasionally cause mycotic aneurysms of the aorta [6,7].

**Figure 3:**
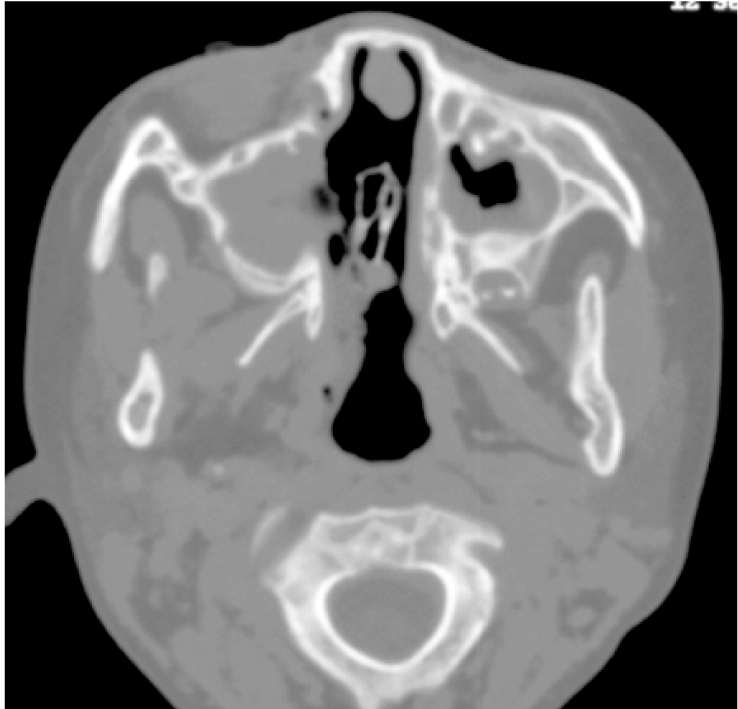
Fungal sinusitis of a four-year-old girl with myeloid leukaemia. CT of the sinuses revealed opacification of the maxillary sinuses with bone destruction of the medial wall. Mucor species was isolated from maxillary sinus washout.

The other main route of Aspergillus into the body is via the sinuses. This can cause a sinusitis that may become aggressive and invade blood vessels, resulting in osteomyelitis and bone destruction of the walls of the sinus. Bone destruction of the walls of the maxillary antrum was noticed in two out of three patients with sinus disease (Figure 3). Fungi were isolated from the sinus wash out of these two patients. Invasive central nervous system aspergillosis is occurring with increased frequency [8], resulting in meningitis, abscess, or encephalitis although none of these features were seen in the current series. One child had lesions in the brain, but the nature of the lesions was never elucidated.

Another fungal infection is caused by the mucormycoses, which include Mucor. Like Aspergillus, Mucor can cause vascular invasion resulting in infarction. The sinuses are the most common site of infection, and this was seen in one patient in our series. Involvement of the lungs can result in consolidation and nodules that may cavitate.

**Figure 4:**
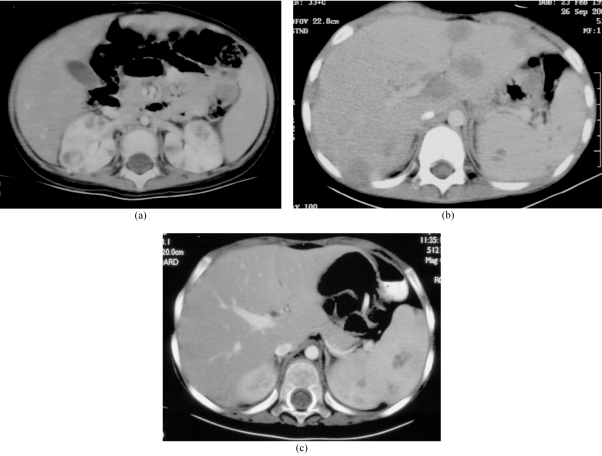
Fungal abscesses in intrabdominal organs. (a) A two-year-old girl with leukaemia and Aspergillus sepsis. CT showed multiple hypodense lesions in both kidneys. (b) A six-year-old girl with leukemia and presumed fungal sepsis. CT revealed multiple hypodense lesions in the liver and spleen. Fungal elements were isolated from liver biopsy, but the species could not be identified. (c) A three-year-old boy with leukemia with presumed fungal sepsis. CT abdomen showed multiple hypodense splenic lesions No fungi were isolated from biopsy.

Besides the lungs and the sinuses, fungal micro-abscesses may be seen in the liver, spleen, and kidneys (Figure 4). These are most commonly caused by Candida, but may also occur due to Aspergillus. Among the three patients with multiple low attenuation areas in the liver, spleen, or kidneys on CT, Aspergillus was isolated in one patient and unidentified fungal elements were isolated in another. But, in the third, there were no isolates.

Other pathological fungi, such as, Zygomycetes, Trichosporon, and Fusarium are becoming recognised [9,10], and some of these may be resistant to the anti-fungal agent Amphotericin.

Because of the need for early treatment, subsequent isolation of the fungus may be difficult [11]. Because of the controversy over the diagnostic criteria for opportunistic invasive fungal infection, an American-European group has developed standard definitions for fungal infections and proposed three levels of probability: proven, probable, and possible [12]. Empirical anti-fungal treatment is usually commenced in patients after 4-6 days of persistent febrile neutropenia not showing a response to maximum antibiotic treatment. The anti-fungal agent of first choice is Amphotericin B because of its proven broad spectrum efficacy, ideally in a lipid formulation. Occasionally, Itraconazole is used as the first choice anti-fungal agent if the renal function is impaired and the patient did not develop the fungal infection while receiving itraconazole prophylaxis. If a fungal infection is proved, treatment with anti-fungal agents should be continued for several months.

**Figure 5:**
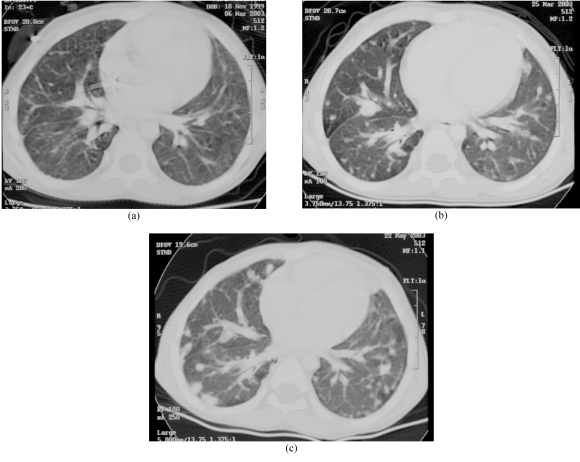
A four-year-old boy with sacrococcygeal endodermal sinus tumour. Blood culture grew *Peiciliomyces lilacinus*. (a) CT lungs showed tiny nodular lesions, presumed involvement of lungs by fungus. (b) After two weeks of anti-fungal therapy, CT showed the lung lesions had increased in size and number. (c) CT lungs after two months of continuous anti-fungal therapy showed more and larger lesions, which were likely to be metastases.

Pulmonary infection counts for 70% of all fatal complications in patients treated for acute leukaemia [13]. One difficulty in diagnosing pulmonary fungal infections radiologically in immune compromised patients is caused by the multiplicity of pulmonary disorders that may occur. Besides fungal infections, there may be pneumonia due to viral, bacterial, or protozoal organisms, adult respiratory distress syndrome (ARDS), infarction, and leukaemic infiltration. In patients with leukaemia, lung haemorrhage occurs and this has been thought to be the sole cause of pulmonary shadowing in as high as 40% of leukaemic patients [14]. Other lung changes may occur with neoplastic involvement (Figure 5) and due to the complications of therapy. Sometimes no cause of the pulmonary shadowing can be found, and it may be necessary to resort to lung biopsy. Through biopsy, a diagnosis of interstitial pneumonitis or organising pneumonia can be made. In our series, seven patients showed lung consolidation on imaging and fungi was identified in only three of these patients.

A study to assess the accuracy of the plain chest radiograph in diagnosing pulmonary complications of leukaemia in children was undertaken by Winer-Muram and her colleagues [13], who compared findings on portable chest radiographs with subsequent post-mortem findings. The accuracy for diagnosing fungal pneumonia was only 55% and they attributed this poor diagnostic accuracy to the variable radiographic appearance of fungal pneumonia and the absence of characteristic radiographic features.

Similarly, in the abdomen, fungal infection can cause low attenuation areas on CT in the liver, spleen, or kidneys, which may be mimicked by metastases, lymphoma, pyogenic abscesses, and tuberculosis. The diagnosis may be difficult because blood cultures may be negative and the symptoms non-specific [15].

The safe environmental level of fungal counts has not been standardised for immune compromised patients. Rhame [16] found that outside, unfiltered air averages 1-15 colony forming units/m3 (cfu/m3), while highly protected hospital areas with air filtered at high efficiency had counts as low as 0.01 cfu/m3. Arnow *et al* [17] found an infection rate of about 3.5% with Aspergillus count levels of 1-2 cfu/m3. The situation described in the present account had fungal counts many times in excess of these values.

A similar cluster of fungal infections caused by released fungal spores from dormant soil reservoirs, due to adjacent construction work, was reported by Leng *et al* [18] when eight patients with leukaemia were diagnosed with invasive sinusitis, although none were presented with concomitant pulmonary involvement. Aspergillus was isolated from seven of these patients. Although the report is restricted to patients with sinus disease, it was noted that the incidence of invasive fungal bronchopulmonary disease was also high in the same ward. Air cultures during the construction period frequently grew Aspergillus and the authors advocated increased precautions against airborne pathogens when susceptible patients are treated. Steifel *et al* [19] found that when a seven-story building was demolished and fungal counts outdoors increased, with preventive measures to seal doors and windows and manipulation of the air handling system, there was no significant rise in fungal counts in nearby hospital wards.

In the present situation, fungal spore counts were performed when the high incidence of fungal infections in the leukaemic children and those with other malignant diseases was recognised. The high spore counts were attributed to release of spores due to adjacent construction work for a new hospital block. The best way to prevent infection was to reduce the air contamination by installing a filter or by moving the patients to a clean area. Neither solution was logistically possible. Measures, however, were taken to identify high-risk patients. Because of their intensive chemotherapy regimen and subsequent prolonged neutropenia, patients with acute myeloblastic and lymphoblastic leukaemia, neuroblastoma, and non Hodgkin’s lymphoma were considered at high-risk for fungal infection. Admission was discouraged in patients with these conditions and the risks explained. The high- risk patients were given itraconazole prophylaxis and also granulocyte colony stimulating factor (GCSF) to reduce the degree and the duration of neutropenia induced by chemotherapy. These measures were not highly successful as evidenced by fungal infection occurring while the patients were on anti-fungal prophylaxis.

Fungal infection should be considered after four to six days of febrile neutropenia. Sometimes the abnormal findings on imaging investigations do permit a diagnosis of fungal disease, but even if a specific diagnosis cannot be reached, imaging can monitor the response to treatment and detect any further complications. To conclude, imaging remains important for patients undergoing chemotherapy for leukaemia and other malignant diseases.
